# Sclerosing Angiomatoid Nodular Transformation of the Laparoscopically Resected Spleen: Case Reports and Review of the Literature

**DOI:** 10.70352/scrj.cr.24-0057

**Published:** 2025-04-02

**Authors:** Shingo Yamasaki, Hiroto Nishino, Takayuki Anazawa, Yuki Teramoto, Takahiro Nishio, Shoichi Kageyama, Kazuyuki Nagai, Yoichiro Uchida, Hironori Haga, Etsuro Hatano

**Affiliations:** 1 Department of Surgery, Graduate School of Medicine, Kyoto University, Kyoto, Kyoto, Japan; 2 Department of Diagnostic Pathology, Kyoto University Hospital, Kyoto, Kyoto, Japan

**Keywords:** splenectomy, sclerosing angiomatoid nodular transformation, EUS-FNA

## Abstract

**INTRODUCTION:**

Most splenic tumors are benign; however, it is essential to differentiate them from malignant tumors, such as malignant lymphoma and metastatic tumors. Sclerosing angiomatoid nodular transformation (SANT) is a relatively rare benign tumor that has been reported recently. Splenectomy is performed in most cases of SANT because of the challenges associated with a definitive diagnosis. However, in some cases, SANT can be diagnosed through endoscopic ultrasound-guided fine-needle aspiration (EUS-FNA), and these cases are subsequently followed up. In this report, we present 2 cases of splenic SANT that underwent laparoscopic resection. In Case 1, atypical imaging findings required EUS-FNA for further evaluation. Case 2 exhibited typical imaging findings of SANT, and therefore, EUS-FNA was not performed.

**CASE PRESENTATION:**

Case 1: A 47-year-old female was found to have a 26 mm tumor in the spleen on abdominal ultrasonography during follow-up for gallbladder polyps. Abdominal computed tomography (CT), magnetic resonance imaging (MRI), and positron emission tomography-CT were performed. EUS-FNA was performed because of the high surgical risk associated with pulmonary hypertension and because hemangioendothelioma, an intermediate malignancy, was suspected. Subsequently, laparoscopic splenectomy was performed, and SANT was diagnosed. Case 2: A 46-year-old female had an incidental detection of a tumor in the spleen on CT. SANT was suspected based on CT and MRI findings. Malignancy could not be completely ruled out owing to the gradual growth of the mass; therefore, the patient was referred to our hospital for surgery. Laparoscopic splenectomy was performed, and SANT was subsequently diagnosed.

**CONCLUSION:**

SANT is a benign tumor that is difficult to diagnose; however, in some cases, it can be diagnosed using EUS-FNA. We report 2 cases of SANT of the spleen that underwent laparoscopic resection. In cases where the diagnosis is confirmed through imaging or histological examination, disease management with follow-up and without surgery is a possible alternative.

## Abbreviations

CT
: computed tomography
ERG
: Ets-related gene
EUS-FNA
: endoscopic ultrasound-guided fine-needle aspiration
FDG
: 18F-fluorodeoxyglucose
MRI
: magnetic resonance imaging
OPSI
: overwhelming post-splenectomy infections
PET/CT
: positron emission tomography/CT
SANT
: sclerosing angiomatoid nodular transformation
SMA
: smooth muscle actin
SUV
: standardized uptake value
T1WI/T2WI
: T1- and T2-weighted images

## INTRODUCTION

Sclerosing angiomatoid nodular transformation (SANT) of the spleen is a rare benign tumor, first proposed by Martel et al. in 2004.^1)^ Prior to this, it was considered a non-tumorous vascular tumor and was often confused with hemangioma. SANT is an isolated tumor with clear borders, consisting of multiple nodules resembling red pulp, with sclerosing inflammatory fibroblasts present between these nodules as a fibrous stroma. Diagnosis is based on the identification of 3 types of vessels by immunostaining: capillaries, sinusoids, and small veins. SANT is difficult to differentiate from other benign tumors, such as hamartoma and hemangioma, and from malignant tumors, such as malignant lymphoma or metastatic tumors, based on imaging findings alone. Therefore, surgery is often chosen for the diagnostic treatment of benign SANT. However, in some cases, SANT can be diagnosed using endoscopic ultrasound-guided fine-needle aspiration (EUS-FNA), and these cases are being followed up. In this report, we describe 2 cases of SANT of the spleen and discuss the possibility of avoiding surgery.

## CASE PRESENTATION

### Case 1

**Fig. 1:**
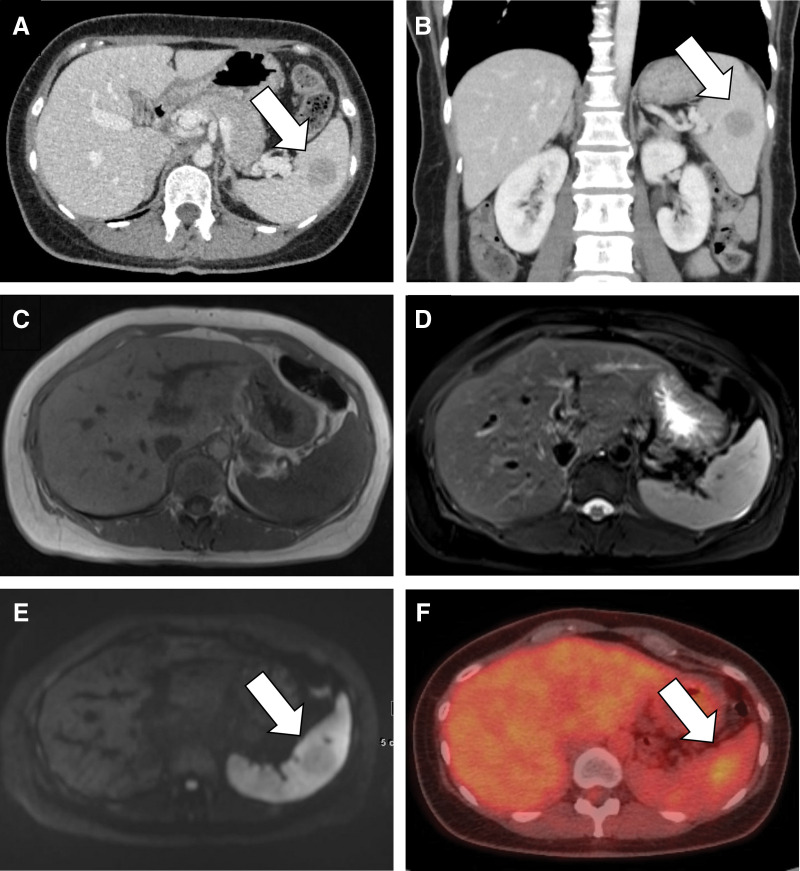
Imaging findings of Case 1. (**A** and **B**) In the venous phase, a 25-mm large round tumor in the spleen, which is lower than the adjacent splenic parenchyma on axial and coronal sections (white arrow), is enhanced on contrast-enhanced CT. (**C**) The tumor is not detected on T1-weighted images of the spleen on MRI. (**D**) The tumor is not detected on T2-weighted images of the spleen on MRI. (**E**) The tumor has a low signal compared to the splenic parenchyma on diffusion-weighted images in the MRI (white arrow). (**F**) PET/CT shows moderate uptake, with SUVmax = 3.0, in the spleen (white arrow). MRI T1/T2 iso-signal with the spleen is seen in SANT, but is also seen in malignant lymphoma and hamartoma. No “spoke-wheel pattern” is indicated on CT. Moderate uptake on PET/CT is observed in SANT. CT, computed tomography; MRI, magnetic resonance imaging; PET/CT, positron emission tomography/CT; SANT, sclerosing angiomatoid nodular transformation

A 47-year-old female was followed-up by a local physician for gallbladder polyps. Abdominal ultrasonography revealed a 26-mm splenic tumor. The patient was receiving medical treatment for pulmonary arterial hypertension (New York Heart Association Classification: II–III) and was referred to our hospital for a detailed examination. The general blood examination results were within normal limits. The serum carbohydrate antigen 125 was 86.2 U/mL (normal range: <20 U/mL), and the brain natriuretic peptide was 53.9 pg/mL (normal range: <18.4 pg/mL). Serum carbohydrate antigen 19-9 and carcinoembryonic antigen, and soluble interleukin-2 receptor levels were within normal limits. Contrast-enhanced computed tomography (CT) detected a 25-mm round tumor in the spleen, which was not identified in the same area on plain CT 1 year and 4 months earlier (**Fig. 1A** and **1B**). Magnetic resonance imaging (MRI) revealed a slightly low signal on diffusion-weighted images and the same signal as the splenic parenchyma on T1- and T2-weighted images (T1WI/T2WI) (**Fig. 1C–1E**). Positron emission tomography/CT (PET/CT) showed moderate uptake (standardized uptake value [SUV]max = 3.0) in the spleen, but no other uptake throughout the body (**Fig. 1F**). Based on the imaging findings, hamartoma, hemangioma, malignant lymphoma, metastatic tumor, or inflammatory pseudotumor was suspected. Although surgery was recommended as a diagnostic treatment, EUS-FNA was performed because the patient had cardiopulmonary risks owing to pulmonary arterial hypertension. EUS revealed a hypoechoic area measuring 30 mm in the spleen, and a biopsy was performed. Histologically, proliferation of spindle-shaped to polygonal cells was observed against a background of fibrous tissue, which is not typical of hamartomas or lymphomas. The tumor cells revealed vacuoles adjacent to the nucleus, and immunostaining was positive for vascular markers such as CD31 and Ets-related gene (ERG). In addition, CD34- and CD8-positive cells were scattered. Although a vascular tumor was suspected, the diagnosis was limited to hemangioendothelioma because of tissue fragmentation and a limited observation area. Hemangioendothelioma is an intermediate malignancy^2)^ in which the tumor is enlarged. The patient underwent surgery for diagnostic purposes. The patient underwent laparoscopic splenectomy with 5 ports in the right semi-lateral decubitus position. The splenic artery and vein were dissected at the splenic hilum using an automatic suturing device, and the spleen was completely resected. The operative time was 2 h and 10 min, with minimal blood loss. After 2 days of cardiopulmonary management using a Swan–Ganz catheter in the intensive care unit, the patient was discharged on the 9th postoperative day with a good course.

**Fig. 2:**
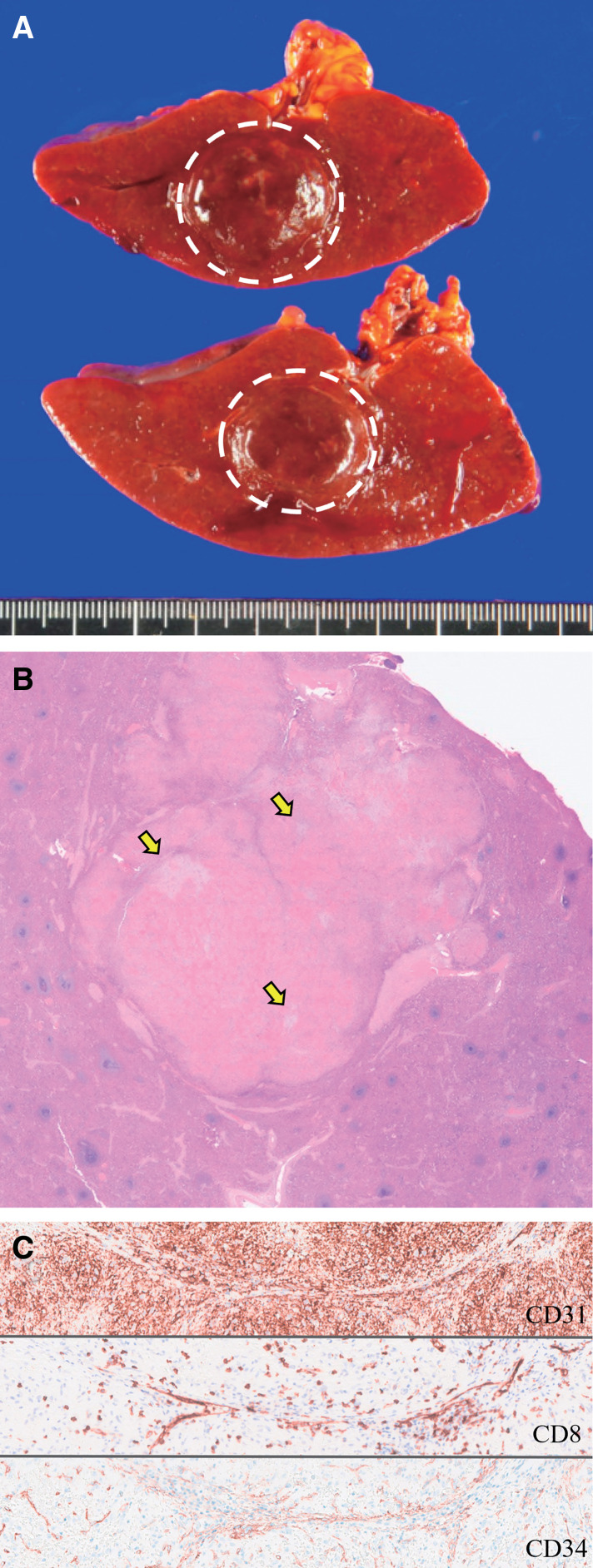
Histopathological findings of Case 1. (**A**) Gross examination of the spleen. A well-defined spherical mass measuring 24 × 24 × 20 mm is observed. (**B**) Low-magnification image showing a multinodular appearance without a capsule. Fibrotic sclerosis is also observed (yellow arrow). (**C**) Immunostaining for CD31, CD8, and CD34 (×20). Three types of vessels are identified: capillaries (CD31+/CD34+/CD8−), sinusoids (CD31+/CD34−/CD8+), and small veins (CD31+/CD34−/CD8−).

Macroscopically, the tumor was a well-demarcated reddish-brown tumor measuring 2.4 × 2.4 × 2.0 cm (**Fig. 2A**). Histologically, the tumor was composed of red nodules of varying sizes with irregular fibrous foci, such as stellate shapes (**Fig. 2B**). The nodules comprised 3 types of blood vessels: capillaries (CD31+/CD34+/CD8−), sinusoidal vessels (CD31+/CD34−/CD8+), and small veins (CD31+/CD34−/CD8−) (**Fig. 2C**). There was no atypia in the vascular endothelium, and no mitotic figures were observed. Based on these findings, the tumor was pathologically diagnosed as a SANT. Retrospectively, it was presumed that the fibrous septum was sampled during EUS-FNA. No atypia or signs of mitosis were observed in the endothelium. Therefore, the final diagnosis was SANT. No recurrence was observed 12 months postoperatively.

### Case 2

**Fig. 3:**
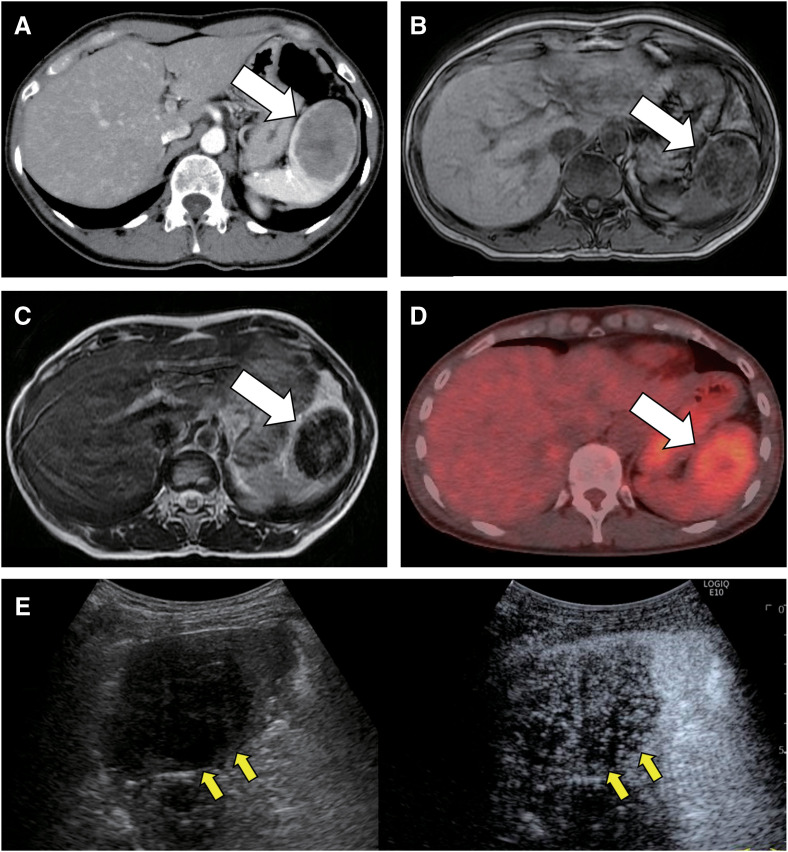
Imaging findings of Case 2. (**A**) A 60-mm large round tumor with clear borders and heterogeneous internal contrast because of central scarring is enhanced on contrast-enhanced CT (white arrow). (**B** and **C**) The tumor is detected with low internal heterogeneity on T1- and T2-weighted MRI (white arrow). (**D**) PET/CT showing moderate uptake (white arrow). (**E**) Sonazoid-enhanced ultrasonography shows a mass with a hypoechoic area. The tumor shows delayed and weak enhancement, with areas showing no heterogeneous enhancement (yellow arrow). Central scarring reflecting fibrous stroma is seen in SANT and hamartoma, but not in hemangiomas. MRI T2 low-signal and moderate uptake on PET/CT are observed in SANT. CT, computed tomography; MRI, magnetic resonance imaging; PET/CT, positron emission tomography/CT; SANT, sclerosing angiomatoid nodular transformation

A 46-year-old female who had undergone surgery for uterine fibroids visited a physician with the chief complaint of cough. During the examination, a splenic tumor was incidentally detected on CT. The patient underwent contrast-enhanced CT, plain MRI, and ultrasonography, and the tumor was suggested to be a SANT based on the imaging findings. However, a CT scan performed 5 years prior did not detect any tumors, and the tumor showed a tendency to enlarge. Malignancy could not be ruled out, and the patient was referred to our hospital for surgery. Blood tests revealed no significant findings, including those for tumor markers. Contrast-enhanced CT detected a 60-mm round tumor in the spleen with clear borders and heterogeneous weak enhancement (**Fig. 3A**). MRI revealed that the mass had low signal intensity with internal heterogeneity on T1WI and low signal intensity on T2WI (**Fig. 3B** and **3C**). PET/CT revealed uptake only in the spleen (**Fig. 3D**). Sonazoid-enhanced ultrasonography revealed a mass in the spleen with a hypoechoic area and irregular septation. The tumor showed delayed and weak enhancement compared to the adjacent splenic parenchyma, with areas showing no heterogeneous enhancement (**Fig. 3E**). Based on these imaging findings and because of the difficulty in ruling out malignancy and the tendency to enlarge, surgical resection of the spleen containing the tumor was decided for diagnostic purposes. The patient underwent laparoscopic splenectomy with 5 ports in the right semi-lateral decubitus position. The operative time was 2 h and 41 min, and the blood loss was 150 g. The postoperative course was uneventful, and the patient was discharged on the 4th postoperative day.

**Fig. 4:**
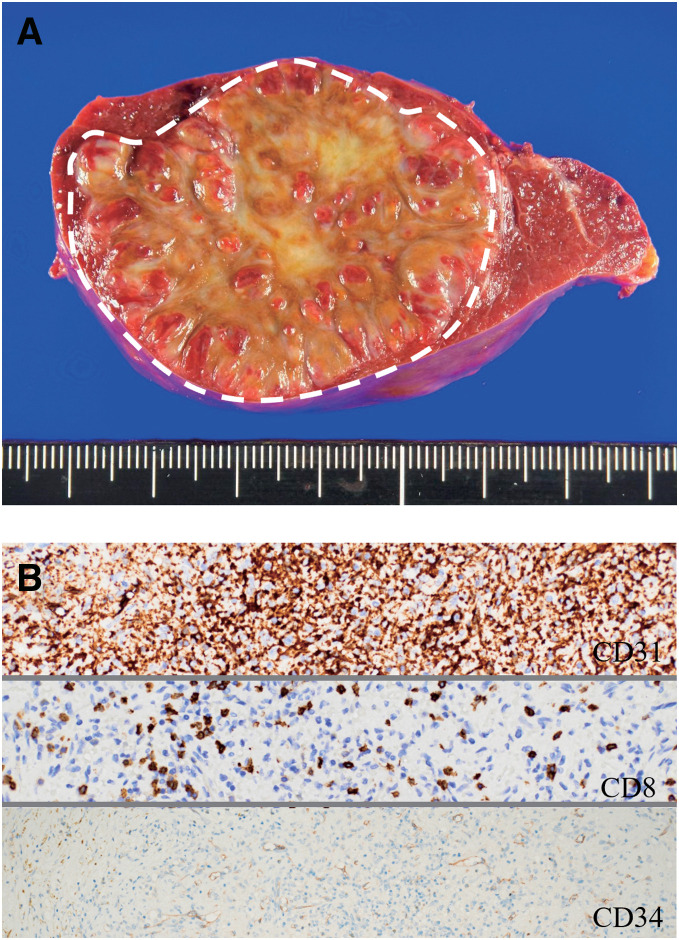
Histopathological findings of Case 2. (**A**) Gross examination of the spleen. A well-defined spherical mass measuring 52 × 51 × 41 mm, with several reddish nodules bordered by fibrous connective tissue inside the mass is observed. (**B**) Immunostaining for CD31, CD8, and CD34 (×20). Three types of vessels are identified: capillaries (CD31+/CD34+/CD8−), sinusoids (CD31+/CD34−/CD8+), and small veins (CD31+/CD34−/CD8−).

Macroscopic examination of the resected specimen revealed a well-defined reddish-brown mass measuring 52 × 51 × 41 mm, with numerous reddish nodules surrounded by fibrous connective tissue inside the mass (**Fig. 4A**). Microscopic examination revealed that the mass was composed of slit-like to irregular vessels, as in Case 1, and immunostaining revealed that it was composed of 3 types of blood vessels, classified as CD31, CD8, and CD34 (**Fig. 4B**). Therefore, the diagnosis of SANT was confirmed. No recurrence was observed 9 months after surgery.

## DISCUSSION

Pathological analysis revealed that SANT comprises 3 types of blood vessels: capillaries, sinusoids, and small veins. The capillaries in SANT express CD31+/CD34+/CD8−, resembling normal capillaries of the splenic red pulp, and sinusoids are characterized by CD31+/CD34−/CD8+ expression, similar to splenic sinusoids. Small veins demonstrate a CD31+/CD34−/CD8− profile, similar to that of small veins in the normal splenic vasculature. These vascular profiles suggest that SANT represents a reactive rather than neoplastic process, potentially arising as an exaggerated response to an unidentified stimulus. The inflammatory stroma of the lesion, comprising variable lymphoplasmacytic infiltrates, spindle cells, and collagenous tissue, further supports this interpretation. The presence of plasma cells and myofibroblasts indicates an ongoing immune response and chronic inflammation, which probably contributes to stromal remodeling and vessel proliferation. Furthermore, the etiology of SANT remains undetermined. Associations between Epstein–Barr virus infection and IgG4-related diseases have been reported; however, there are conflicting reports, and their definitive relationship remains unclear.^3)^ However, recent reports have indicated that SANT lacks CTNNB1 exon 3, suggesting that it may be preneoplastic or neoplastic.^4,5)^ In addition, the expression of smooth muscle actin (SMA) and CD68 in perinodular spindle cells and fibrous tissues has been reported.^6,7)^

Although there have been a few reports of SANT detection triggered by anemia, thrombocytopenia, and portal hypertension,^8–10)^ SANT is often detected asymptomatically and incidentally on imaging. SANT can be classified as a solitary solid lesion,^11)^ which also includes hemangioma, hamartoma, inflammatory pseudotumors, and malignant tumors, such as angiosarcoma and lymphoma. In particular, many non-hematopoietic and non-vascular neoplasms and pseudoneoplastic lesions have pathologically mixed fibrotic and inflammatory components, which explains why they are indistinguishable on imaging.^12)^

Imaging findings typically include enhancement of the arterial phase on contrast-enhanced CT, which is characterized by enhancement that begins peripherally and progresses gradually radially toward the center. In addition, a characteristic imaging feature known as the “spoke-wheel pattern” may be observed when fibrous stroma is depicted as a non-enhancing area, extending radially toward the center of the lesion. On MRI, the tumor often appears as an area of low-signal intensity on T2WI.^13,14)^ PET/CT scans may show mild-to-moderate 18F-fluorodeoxyglucose (FDG) uptake.^15)^ In both of our cases, the characteristic “spoke-wheel” appearance was not observed. However, the imaging findings in Case 1 reflected the gross findings well and showed nearly homogeneous internal findings, whereas Case 2 showed heterogeneous internal findings because of central scarring, which is typical of SANT.

Aziret et al.^16)^ performed a systematic review of 230 SANT cases in the literature; 52.1% were female, and the median age was 46 years (9 weeks–85 years). A total of 166 patients (72.1%) underwent splenectomy: 35 (15.2%) laparoscopic splenectomy, 15 (6.5%) laparoscopic partial splenectomy, 4 (1.7%) open splenectomy, 3 (1.3%) partial splenectomy, and 2 (0.8%) manual laparoscopic splenectomy. In our cases, we performed laparoscopic splenectomy, which is a suitable approach for treating SANTs in the spleen because they typically have clear borders and less potential for invasion into the surrounding area. However, there is a risk of complications owing to splenectomy, particularly the risk of overwhelming post-splenectomy infections (OPSI). Jin et al.^17)^ reported that laparoscopic partial splenectomy for SANT tended to have a longer operative time than laparoscopic splenectomy; however, there was no significant difference in blood loss. Partial splenectomy has the advantage of reducing the risk of OPSI.^18)^ However, complete radical resection is required if malignancy is suspected, as noted in Case 1.

In Case 1, EUS-FNA was performed because of the high surgical risk; however, a definitive diagnosis could not be made. This may be because tissue containing multiple nodules and 3 different types of vascular components, which are characteristics of SANT, was not collected in the biopsy. However, Katsuda et al.^19)^ reported a case in which a definitive diagnosis of SANT was made using EUS-FNA, and the patient was followed up. This indicates that EUS-FNA may be useful for the diagnosis of SANT if sufficient tissue is obtained. Aziret et al.^16)^ reported that biopsies were performed in 8 patients; however, only 3 were definitively diagnosed with SANT. We further summarized the clinical characteristics of the 14 patients until April 2024^19–31)^ (**Table 1**). Of the 14 patients, 9 were diagnosed with SANT on biopsy (64.3%; 1 case was a tentative diagnosis of SANT). Of the 9 patients, 7 (77.8%) were followed up without surgery. Compared with percutaneous needle biopsy, EUS-FNA of the spleen is safe because it allows clear visualization of the spleen and splenic artery.^32)^ Although there is a risk of bleeding from the splenic parenchyma because of the risk of peritoneal dissemination if the tumor is malignant, the risk of pancreatic cancer by EUS-FNA is reported to be relatively safe at 3.8%.^33)^ Therefore, EUS-FNA may be a useful option in cases of high surgical risk or when SANT is clinically suspected. In some of the case reports summarized in **Table 1**, punctures were repeated. In Case 1, the patient underwent surgery because SANT was not suspected based on imaging findings; however, if SANT was suspected, several biopsies may be considered.

**Table 1:** Summary of previously reported cases in which biopsies were performed for SANT

Author	Year	Sex	Age	Symptom	Solitary or multiple	Size of tumor	Kind of biopsy	Result of biopsy	Operation	Clinical course
Weinreb et al.^20)^	2007	F	41	None	Solitary	65 mm	Core needle biopsy	A tentative diagnosis of SANT	OS	No recurrence in1 month
Gutzeit et al.^21)^	2009	M	77	None	Solitary	80 mm	Core needle biopsy (16G)	SANT	None	No data
Sitaraman et al.^22)^	2010	M	65	None	Solitary	20 mm	EUS-FNA	No diagnosis	OS	No data
Kim et al.^23)^	2012	F	43	None	Solitary	20 mm	No data	SANT	None	No recurrence in 13 months
Murthy et al.^24)^	2015	M	56	Left upper quadrant pain and mild anemia	Solitary	100 mm	Core needle biopsy	Paucicellular fibrosis with medium-caliber vessels embedded in fibrous tissue, showing signs of obliteration and/or recanalization	OS	No data
Dutta et al.^25)^	2016	F	60	None	Solitary	38 mm	1st FNA	1st: normal spleen	No data	No data
							2nd Tru-Cut biopsy	2nd: SANT		
Demirci et al.^26)^	2017	M	43	None	Multiple	35 mm, 17 mm	FNA	1st FNA was inconspicuous. 2nd one contained atypical, hyperchromatic, and polymorphic cells	LS	No recurrence
Sharma et al.^27)^	2018	F	56	None	Solitary	38 mm	FNA	SANT	None	No recurrence in 1 year
Sánchez Belmar et al.^28)^	2020	M	42	None	Solitary	85 mm	FNA	Presented evidence of infarction and inflammation	OS	No data
Papatheodrou et al.^29)^	2021	F	55	Abdominal pain	Solitary	32 mm	Under-CT biopsy	SANT	LS	No recurrence in 8 months
Katsuda et al. ^19)^	2021	M	72	None	Solitary	34 mm	EUS-FNA (25G)	SANT	None	No data
Beyhan et al.^30)^	2024	F	28	Abdominal pain	Multiple	No data	No data	SANT	No data	No data
Gómez Rubio et al.^31)^	2024	M	50	None	Solitary	20 mm	FNA (19.5G)	SANT	None	No recurrence in 10 years
Our case		F	47	None	Solitary	25 mm	EUS-FNA (22G)	Spindle-shaped cells	LS	No recurrence in 12 months

CT, computed tomography; EUS-FNA, endoscopic ultrasound-guided fine-needle aspiration; F, female; LS, laparoscopic splenectomy; M, male; OS, open splenectomy; SANT, sclerosing angiomatoid nodular transformation

Tseng et al.^34)^ aimed to raise awareness of this emerging diagnosis of SANT, which is currently categorized as a rare disease but will hopefully be more commonly recognized with time among clinicians and surgeons. They recommended follow-up of patients diagnosed with SANT using imaging. If a definitive diagnosis can be made through biopsy, follow-up may be an option. SANT can be suspected if a “spoke-wheel pattern” or “central scar” is observed on imaging. In the absence of these findings, septal or mottled contrast effects may indicate SANT. Although distinguishing SANT from hamartomas and inflammatory pseudotumors with similar pathological backgrounds is challenging, EUS-FNA can be considered for tumors suspected to be SANT. In Case 2, EUS-FNA could have been performed, potentially avoiding surgery if a diagnosis had been made. Further accumulation of such cases is required.

## CONCLUSION

SANT is a benign tumor that is challenging to diagnose because of the absence of characteristic clinical findings. Splenectomy is a useful strategy for the diagnosis and treatment of SANT. However, if the diagnosis is confirmed through imaging or histological findings from biopsy and FNA, particularly EUS-FNA, management of the disease with follow-up and without surgery can be a possible alternative.

## ACKNOWLEDGMENTS

We would like to thank Editage (www.editage.jp) for the English language editing.

## DECLARATIONS

### Funding

Not applicable.

### Authors’ contributions

SY wrote the draft of the manuscript.

HN and TA revised the manuscript.

YT described the pathological findings of the resected specimen and revised the manuscript.

All authors read and approved the final manuscript and are accountable for all aspects of the work.

### Availability of data and materials

The datasets supporting the conclusions of this article are included within the article.

### Ethics approval and consent to participate

We conducted our study in accordance with the Declaration of Helsinki.

### Consent for publication

Written informed consent was obtained from the patient for publication.

### Competing interests

The authors declare that they have no competing interests.
